# Estrogen-Receptor Expression and Function in Thymocytes in Relation to Gender and Age

**DOI:** 10.1155/1998/62380

**Published:** 1998

**Authors:** F. Kohen, L. Abel, A. Sharp, Y. Amir-Zaltsman, D. Sömjen, S. Luria, G. Mor, A. Knyszynski, H. Thole, A. Globerson

**Affiliations:** 1 Department of Biological Regulation The Weizmann Institute of Science Rehovot 76100 Israel; 2 Department of Immunology The Weizmann Institute of Science Rehovot 76100 Israel; 3 Department of Biological Services The Weizmann Institute of Science Rehovot 76100 Israel; 4 Endocrine Unit Tel Aviv Medical Center and the Sackler Faculty of Medicine Tel Aviv University Tel Aviv Israel; 5 Obstetrics and Gynecology Kaplan Hospital Rehovot Israel; 6 Max-Planck-Institut für Experimentelle Endokrinologie Hanover Germany

**Keywords:** Aging, estrogen receptor, thymocytes

## Abstract

The expression of estrogen receptor (ER) in thymocytes was studied in young, middle-aged, and old (2, 12, and 24 months, respectively) female and male C57BL/6J mice. Western immunoblots prepared from the thymocytes of females of all age groups showed the presence of a 67-kD protein band, which has been associated with the apparent MW of denatured ER. Flow cytometry analysis o,f cells stained with a monoclonal anti-ER antibody (clone 13H2) disclosed ER expression in both females and males of all age groups. *In vivo* treatment with estradiol (E_2_) led to an increase in the specific activity of thymic creatine kinase (CK) in the female mice, whereas the male thymocytes responded with an increase in CK activity only on treatment with dihydrotestosterone (DHT). The data show no differences in ER expression between male and females, but the receptor appears not to be functional in males. Interestingly, when estradiol was applied to co-cultures of lymphoid-depleted fetal thymus (FT) explants and bone-marrow cells, or thymocytes, from young and old females, it resulted in increased cellularity of cultures containing cells of the young, and not those of the old. The proportion of CD4/CD8 phenotypes of the developing cells in these cultures was not affected by E_2_ treatment. These observations provide a new insight into ER expression and function in T-cell development in relation to gender and age.


*Developmental Immunology*, 1998, Vol. 5, pp. 277–285
Reprints available directly from the publisher
Photocopying permitted by license only

© 1998 OPA (Overseas Publishers Association)
N.V. Published by license under the
Harwood Academic Publishers imprint,
part of the Gordon and Breach Publishing Group
Printed in Malaysia

### Estrogen-Receptor Expression and Function in Thymocytes in Relation to Gender and Age

F. KOHEN[a*], L. ABEL[b], A. SHARP[c], Y. AMIR-ZALTSMAN[a], D. SÖMJEN[d], S. LURIA[e], G. MOR[a], A. KNYSZYNSKI[b], H. THOLE[f] and A. GLOBERSON[b]

[a]*Department of Biological Regulation,* [b]*Department of Immunology,* [c]*Department of Biological Services the Weizmann Institute of Science, Rehovot, 76100, Israel;* [d]*Endocrine Unit, Tel Aviv Medical Center and the Sackler Faculty of Medicine, Tel Aviv University, Tel Aviv, Israel;* [e]*Obstetrics and Gynecology, Kaplan Hospital, Rehovot, Israel;* [f]*Max-Planck-Institut für Experimentelle Endokrinologie, Hanover, Germany*

*(Received 5 March 1997; Revised 18 June 1997; In final form 24 July 1997)*

The expression of estrogen receptor (ER) in thymocytes was studied in young, middle-aged, and old (2, 12, and 24 months, respectively) female and male C57BL/6J mice. Western immunoblots prepared from the thymocytes of females of all age groups showed the presence of a 67-kD protein band, which has been associated with the apparent MW of denatured ER. Flow cytometry analysis of cells stained with a monoclonal anti-ER antibody (clone 13H2) disclosed ER expression in both females and males of all age groups. *In vivo* treatment with estradiol ($E_2$) led to an increase in the specific activity of thymic creatine kinase (CK) in the female mice, whereas the male thymocytes responded with an increase in CK activity only on treatment with dihydrotestosterone (DHT). The data show no differences in ER expression between male and females, but the receptor appears not to be functional in males. Interestingly, when estradiol was applied to co-cultures of lymphoid-depleted fetal thymus (FT) explants and bone-marrow cells, or thymocytes, from young and old females, it resulted in increased cellularity of cultures containing cells of the young, and not those of the old. The proportion of CD4/CD8 phenotypes of the developing cells in these cultures was not affected by $E_2$ treatment. These observations provide a new insight into ER expression and function in T-cell development in relation to gender and age.

*Keywords:* Aging, estrogen receptor, thymocytes

*Abbreviations:*
Estrogen receptor: ER; estradiol: $E_2$; dihydrotestosterone: DHT; creatine kinase: CK

*Corresponding author. Present address: Department of Biological Regulation, The Weizmann Institute of Science, Rehovot, 76100, Israel

### INTRODUCTION

The idea that gonadal steroids play a role in immunological dimorphism has gained support from various studies (Grossman, 1985, 1989; Marchetti et al., 1995; Sthoeger et al., 1988; Besedovsky and del Rey, 1996). Estrogen binding was shown in human peripheral blood mononuclear cells (Danel et al., 1983; Weusten et al., 1986) and in the thymus (Danel et al., 1983; Gulino et al., 1983, 1985; Luster et al., 1984; Marchetti et al., 1984; Weusten et al., 1986). Expression of ER in the thymus was found mainly in the epithelial cells (Luster et al., 1984; Marchetti et al., 1984), suggesting the involvement of estrogen function in T-lymphocyte development. In previous studies, using a variety of experimental criteria, we demonstrated that ER is expressed in thymocytes of young adult female mice and rats (Amir-Zaltsman et al., 1993). Hence, (1) Western blot analysis of thymocytes showed the presence of a 67-kD protein band; (2) Northern blot analysis of poly(A+)-enriched RNA fraction obtained from the thymocytes showed the presence of a transcript of 6.2 kb, corresponding to the size of ER mRNA; (3) immunofluorescence studies using anti-idiotypic antibody clone $1D_5$ that interacts with ER (Mor et al., 1992) showed staining of the thymocyte nuclei; (4) the mitogen-induced proliferative response was reduced in the presence of estradiol; (5) administration of estradiol to immature female rats caused a significant increase in the thymic ER mRNA and Creatine kinase B (CKb) mRNA (Amir-Zaltsman et al., 1993).

The function of estrogen in the immune system has been of particular interest, in view of its relevance to pregnancy (Clarke and Kendall 1994) and autoimmunity (Cutolo et al., 1995). In addition, since generation of T lymphocytes continues throughout the lifespan, a decline in ER expression in the aging thymus may play a role in developmental processes that change with age (Globerson 1994, 1995). However, there is hardly any information on the status of ER in the aging thymus.

The present study was designed to determine whether the expression and function of ER in thymocytes are age- and gender-specific and to examine its role in lymphoid development in the thymus.

### RESULTS

### Expression of ER in Thymocytes

The first series of experiments was conducted to examine the expression of ER in thymocytes of female and male mice, as related to age. We used Western immunoblots and flow cytometry methods, as in our previous report (Amir-Zaltzman et al., 1993).

Western immunoblots prepared from thymocytes of female mice showed the presence of a 67-kD protein band (lane A in Figure 1a), which has been associated with the apparent MW of denatured ER (Greene et al., 1986). This band was observed in thymocytes of the three age groups (lanes B, C, and D in Figure 1a), and was absent when the primary, specific anti-$E_2$ antibody was omitted in the control gel (Figure 1b). The specific anti-$E_2$ antibody stained the 32-kD fragment in the thymocytes of all mice in the group (lanes B, C, and D in Figure 1a) and was absent in the control blot (lanes B, C, D, and E in Figure 1b).

Representative flow cytometry profiles of thymocytes from young and old females show specific staining with the anti-ER antibody and PE-rabbit anti-mouse IgG, as related to the control of second antibody alone. Similar results were obtained with FITC-labeled goat anti-mouse IgG as a second antibody (Figure 2) or when the directly labeled FITC-anti-ER antibody was used (data not shown). Analysis of thymocytes from females and males of the different age groups revealed a similar proportion of $ER^+$ cells in all cases (Table I).

### Response to Gonadal-Hormone Treatment *In Vivo*

Functional manifestation of ER in thymocytes was investigated in hormone-treated mice, by measuring the CK-response studies. Female and male mice of the

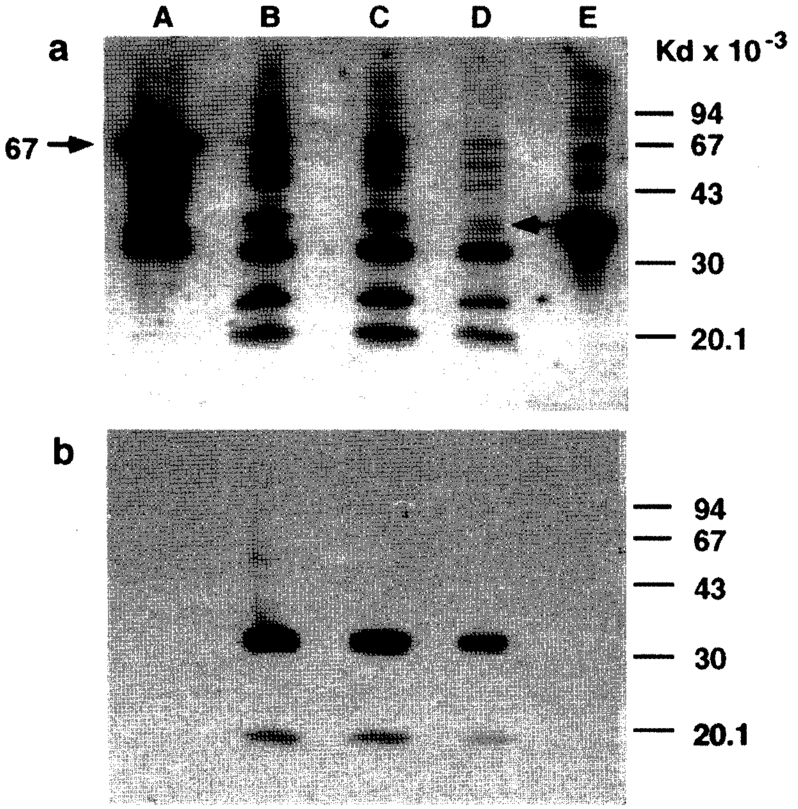

FIGURE 1 Mouse thymocytes (2 × $10^5$ cells/lane), cDNA estrogen-receptor (ER) protein expressed in yeast and the 32-kD purified ER fragment from porcine uteri were subjected to 10% SDS-PAGE and transferred to nitrocellulase and probed first with anti-ER antibody (clone $13H_2$), followed with rabbit anti-mouse $IgG_1$ peroxidase conjugate as described (Amiz-Zaltsman, et al, 1993). The blots were then visualized using enhanced chemiluminescence, and the reagents provided by Amersham. Mol wt markers are shown on the right. (a) Lane A: cDNA ER protein expressed in yeast. Lane B: Mouse thymocytes derived from a 2-month-old C57BL female mouse. Lane C: Mouse thymocytes derived from a 1-year-old C57BL female mouse. Lane D: Mouse thymocytes derived from a 2-year-old C57BL female mouse. Lane E: The 34-kD ER fragment derived from porcine uteri. (b) Controls of lanes A through D probed only with the second antibody rat anti-mouse $IgG_1$ peroxidase.

different age groups were injected with either $E_2$, DHT, or PBS, respectively. Analysis of CK response to hormonal treatment was performed on the intact thymus tissue, to correlate with previous studies (Marchetti et al., 1984), as well as on separated thymocytes, leading to similar results. Figure 3 shows representative results obtained on intact thymus tissue. Hence, CK levels were increased in females treated with $E_2$ and not with DHT, whereas the males showed a response to DHT and not to $E_2$. The sex-specific-induced increase in CK response in the thymus (Figure 3) and in isolated thymocytes (data not shown) was observed in all age groups.

### Effects of $E_2$ on Thymocytopoiesis *In Vitro*

To find out if ER expression plays any role in thymocytopoiesis, we seeded thymocytes and bone-marrow cells, from young and old female mice, onto individual lymphoid-depleted FT lobes. $E_2$ was applied for the first 3 days of cell seeding onto the FT lobes (under the "hanging-drop" conditions), or during the subsequent organ culture period, or throughout the entire *in vitro* period. Control cultures were set up in parallel, without $E_2$. The cultures were sacrificed after 7 days, and cell numbers were counted. In addition, cells were analyzed for CD4/CD8 phenotypes, using a direct double-staining procedure. The results showed a significant increase in numbers of cells originating from young donors, following incubation with $E_2$ during the seeding phase of the culture. No effect was observed in any of the cultures of old donor cells (Table II). On the other hand, the proportions of CD4/CD8 phenotypes were not affected by $E_2$ treatment (Table III).

### DISCUSSION

Expression of ER in the thymus has been demonstrated in the past; however, there has been little information on its expression and function in aging. Our results provide new information regarding ER in relation to gender and age. Whereas ER is expressed in both females and males, only female cells respond to estrogen. Regarding aging, we observed no significant age-related decline in ER expression, as revealed from Western immunoblots, flow cytometry, and CK response. It thus appeared that ER continues to function in advanced age. However, experiments designed to determine if ER expression in aging plays a role in thymocytopoiesis showed that $E_2$ treatment resulted in cellular expansion in the young, but not in the old donor-derived thymocytes.

The finding that thymocytes of male mice express the ER, with no increase in CK activity in response to treatment with $E_2$, suggests that the ER in males is not functional. On the other hand, the male thymocytes responded to DHT, indicating the presence of functional androgen receptors in this organ. These findings are in accordance with the previous results on skeletal cells, where $E_2$ caused specific stimulation of CK activity only in female- and not in male-derived skeletal cells, whereas the male cells responded to

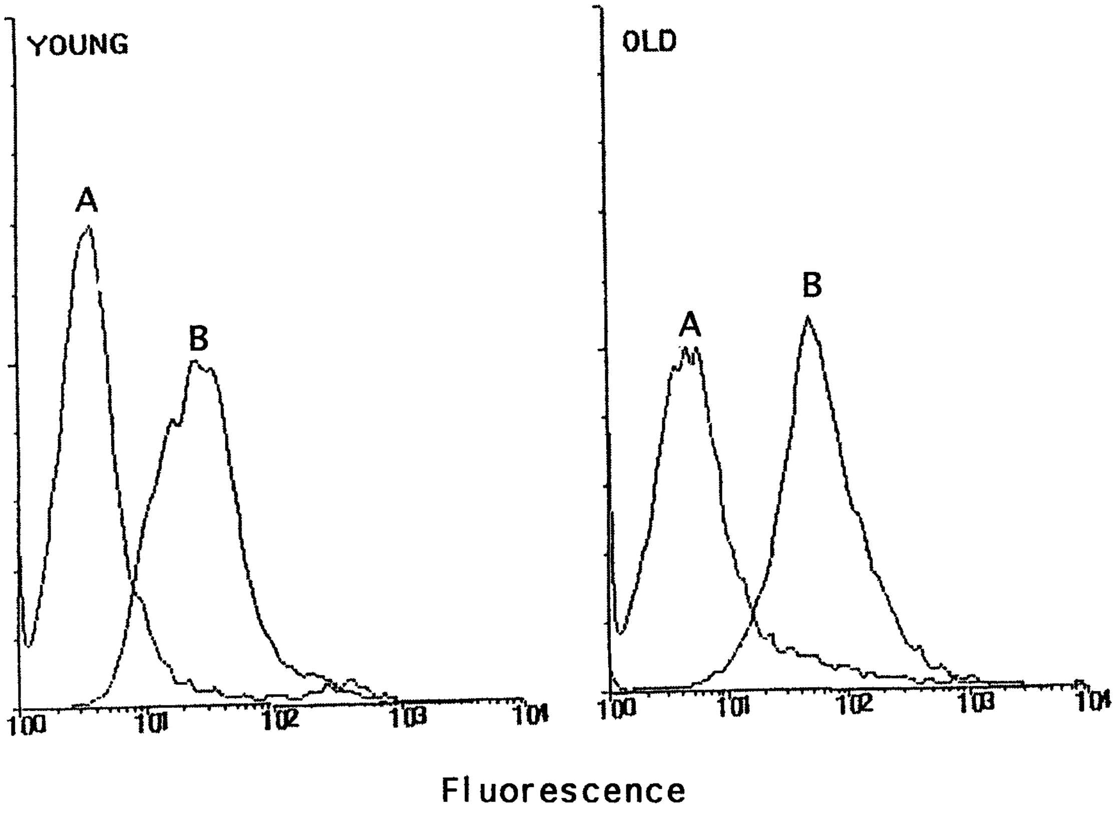

FIGURE 2 Flow cytometry profiles of $ER^+$ thymocytes of young (2 months) and old (24 months) C57BL/6J females. (Curve A) Background control cells stained with the second antibody only. (Curve B) Thymocytes stained in a two-step procedure, using monoclonal anti-ER antibodies ($13H_2$ clone) and PE-labeled rabbit anti-mouse IgG. *Y* axis: cell number (arbitrary units).

TABLE I Flow Cytometry Analysis of ER Expression in Thymocytes of Females and Males of Different Age Groups

| | ER$^+$ cells (%)[a] | |
|---|---|---|
| Age (months) | Females' | Males |
| 2 | 68 ± 15 | 59 ± 12 |
| 12 | 67 ± 9 | 64 ± 10 |
| 24 | 72 ± 7 | 74 ± 6 |

[a]Values represent mean ± SD of percent ER+ thymocytes from six individual mice per group, calculated from two-step staining with an anti-ER monoclonal antibody ($13H_2$) and subsequently with a second step of either FITC-goat anti-mouse or RPE-Rabbit anti-mouse IgG. Mice of all the age groups were examined in parallel in each of three independent experiments.

DHT (Sömjen et al., 1995). Interestingly, it was recently shown that estrogen resistance caused by a mutation in the ER gene in a 28-year-old man resulted in osteoporosis, implicating a role of ER in males (Smith et al., 1994). Our finding that ER is expressed in males, yet, it is not functional in response to $E_2$, as manifested in the CK assay, raises the question of whether it has other possible functions.

Estrogens influence many developmental and physiological responses in target cells by regulating specific gene activity (Parker, 1993). Expression of the receptor in thymic epithelial cells may play a role in processes of stromal-cell induction of lymphocyte development, whereas expression in the thymic lymphoid cells also suggests direct hormonal effects on these cells. Estrogen may thus affect various types of processes in the thymus, including cell division, differentiation, and apoptosis, either directly or via stimulation of cytokine function. Our present study reveals a role of ER on cell division, and no effect on thymocyte subset differentiation, as indicated from the results on young donor cells. The observation of no effect on cell division in case of the old may be attributed to downstream processes that decrease in aging (Globerson, 1995). The mechanisms underlying the functional manifestation and the immunological relevance of the receptor in thymocytes, as well as the nature of the ER in males, will need to be further elucidated.

### MATERIALS AND METHODS

### Mice

Young (2 to 3 months), middle-aged (12 months), and old (24 months) female and male C57BL/6J (Jackson Laboratories, Bar Harbor, ME) and BALB/c mice (OLAC, UK) were used throughout the study. The mice were virus- and pathogen-free (SPF), bred in isolators, and maintained in Millipore-top cages, on sterile bedding, with food and water *ad libitum*. Cleaning and handling were performed under laminar flow hoods. The mice were routinely monitored for possible viral, bacterial, or parasitic contaminations. Only mice with no overt malignancy or any other gross pathological manifestation were included in the study.

### Western Blot Analysis

Mouse thymocytes ($2 \times 10^5$ cells/lane), the 32-kD ER fragment purified from porcine uteri (Thole et al., 1991) and the cDNA ER protein expressed in yeast (Greene et al., 1986) were solubilized in buffer containing 0.07 M TrisHCl, pH 6.8, 10% glycerol, 1% SDS, $\beta$-mercaptoethanol, and bromophenol blue by heating at 100°C for 5 min, and processed for Western blot analysis as described previously (Amir-Zaltsman et al., 1993).

### Antibodies

Mouse monoclonal antibody, clone $13H_2$, raised against porcine 32-kD hormone-binding estrogen receptor (ER) fragment (Thole et al., 1991; Thole and Jakob, 1993) was prepared as described previously. This antibody was conjugated with FITC for direct staining of thymocytes in flow cytometry, using conditions previously described (Mor et al., 1992). Rabbit anti-mouse $IgG_1$ peroxidase and FITC-labeled goat anti-mouse IgG were from Zymed Lab. (South San Francisco) and rabbit anti-mouse RPE, affinity-

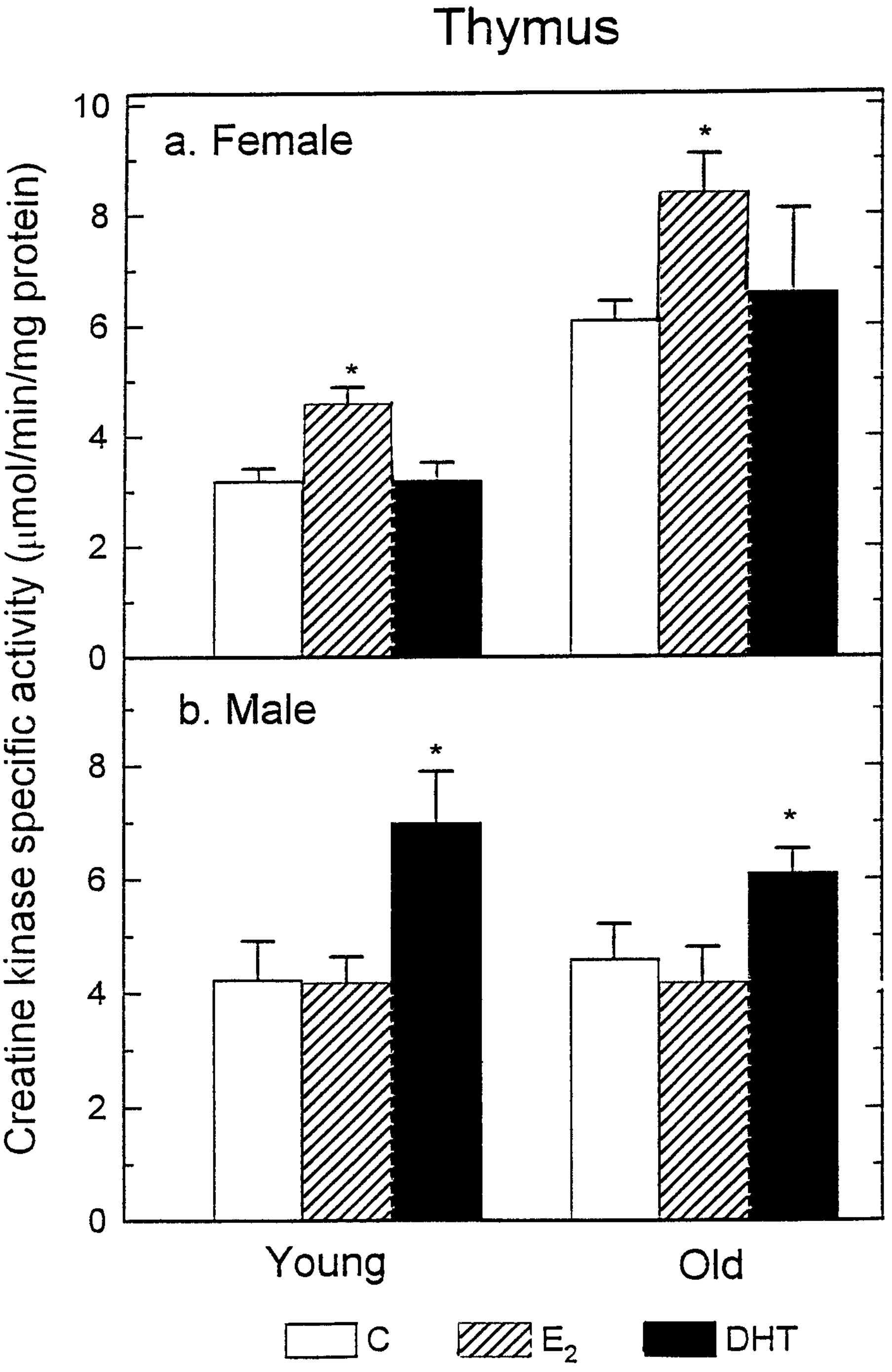

FIGURE 3 Hormonal stimulation of CK-specific activity in mouse thymus. Young and old, female and male mice were injected with $E_2$, DTH, or PBS, as described in Materials and Methods. The thymus of each mouse was then assayed for CK-specific activity. Results are expressed as mean ± S.E.M.; $n = 5$; $*P \leq 0.05$.

TABLE II Effect of $E_2$ on *In Vitro* T-Cell Development from Cells of Young and Old Mice

| Donor cells | Age group | *n* | Cells/lobe × $10^3$ $E_2$ | Control | *P* value |
|---|---|---|---|---|---|
| Thymocytes[a] | Young | 13 | 46.0 ± 3.0 | 31.4 ± 3.0 | 0.005 |
| | Old | 13 | 27.7 ± 2.5 | 29.9 ± 4.4 | NS |
| Bone marrow | Young | 8 | 45.1 ± 7.4 | 30.8 ± 4.2 | 0.02 |
| | Old | 8 | 27.8 ± 3.5 | 29.8 ± 6.0 | NS |

[a]PNA+ thymocyte populations, representing immature cells.
*Note*: Thymocytes, or bone-marrow cells, from young and old mice, were co-cultured with lymphoid depleted fetal thymus lobes.
Data represent mean ± SE values calculated from data obtained in independent experiments (*n*). Each experiment included sets of cultures with cells from individual young and old donor mice.

TABLE III CD4/CD8 Subsets in Co-Cultures of FT and Bone-Marrow Cells from Young and Old Donors Treated with $E_2$[a]

| Subset | $E_2$ | Young control | $E_2$ | Old control |
|---|---|---|---|---|
| $CD4^+CD8^-$ | 10.1 ± 0.7 | 12.2 ± 5.4 | 8.3 ± 1.9 | 6.2 ± 1.8 |
| $CD4^-CD8^+$ | 12.5 ± 2.2 | 13.0 ± 0.8 | 22.0 ± 3.1 | 22.8 ± 5.9 |
| $CD4^+CD8^+$ | 10.6 ± 1.8 | 8.1 ± 2.0 | 14.6 ± 6.6 | 14.5 ± 4.4 |
| $CD4^-CD8^-$ | 66.3 ± 4.5 | 69.0 ± 5.4 | 51.3 ± 6.7 | 56.4 ± 8.8 |

Mean ± SE values of five independent experiments.
*Note*: BM cells were seeded onto irradiated FT explants 1 hr after exposure. $E_2$ was applied during the first 2 days in hanging-drop cultures. Cells were harvested for analysis after 7 days in organ cultures.

isolated $F(ab-)_2$ conjugate was from Dako (Denmark). Anti-mouse CD4 conjugated with PE and FITC-anti-mouse CD8 (Serotec, UK) were used in direct double staining.

### Hormonal Treatment *In Vivo*

The changes in creatine kinase (CK) specific activity in the thymus induced by the short treatment of estradiol ($E_2$), dihydrotestosterone (DHT), or phosphate-buffered saline (PBS, control) were studied in male and female mice of the three age categories. The animals (five mice/group) were sacrificed 24 hr after i.p. injection of $E_2$ (5 $\mu$g/animal), DHT (10 $\mu$g/animal), or PBS.

### Hormonal Treatment *In Vitro*

Organ cultures were prepared as originally described (Eren et al., 1988). Briefly, fetal (day 14 of gestation) thymus (FT) lobes were depleted of lymphoid cells by treatment with 2-deoxyguanosine (1.35 mM) for 5 days at 37°C (Jenkinson et al., 1982), or by exposure to irradiation (20 Gy; Fridkis-Hareli et al., 1991), as specified. The FT lobes were subsequently incubated with the donor cells (60 × $10^3$ cells/lobe), in hanging drops, in Terasaki plates (Nunclon; Denmark), for 3 days; then rinsed and cultivated in organ cultures for 7 days. Donor cells included bone marrow, or immature (PNA+) thymocytes, prepared in accordance to standard procedures (Reisner et al., 1976). Hormonal treatment ($10^{-7}$ M $E_2$) to the cultures was applied either in the hanging drops, or in the organ cultures, or throughout the *in vitro* incubation period.

### Flow Cytometry Analysis

Thymocytes, prepared from each mouse separately (2 × $10^6$ cells/pellet), were fixed in 50% ethanol at −20°C for 10 min. The cells were stained directly (with FITC-anti-ER), or in a two-step procedure, using PE-labeled rabbit anti-mouse IgG as the second antibody. Incubation with the $13H_2$ monoclonal antibody (unlabeled, or FITC-conjugated; 20 $\mu$l from a stock solution of 100 $\mu$g protein/ml PBS containing

0.1% BSA and 5% fetal calf serum) was carried out at 4°C for 45 min. Staining with the flurochrom-conjugated anti-mouse IgG (20 $\mu$l of the stock solution) was under similar conditions. Cells incubated with the second antibody alone served as negative control. Flow cytometry was performed on a FACScan (Becton-Dickinson, Mountain View, CA), using the PCLYSYS II program for analysis of the data.

### Analysis of CK Activity

Analysis of CK activity was carried out on the intact thymus tissue, thymocytes, or thymic stromal tissue; stored at −20°C and processed as previously described (Sömjen et al., 1995).

### Data Analysis

Experimental groups consisted of five mice. Data present were mean ± SE of at least two independent experiments, as specified. Statistical analysis was based on Student's *t* test.

### *Acknowledgements*

We are grateful to Professor G. Greene for the cloned 4ER cDNA expressed in yeast and to Mrs. M. Kopelowitz for excellent secretarial assistance. A.G. is the incumbent of the Harriet and Harold Brady Chair for Cancer research.

This work has been partially funded by the Sandoz Foundation for Gerontological Research, Brussels, Belgium (to A.G.).

### *References*

Amir-Zaltsman Y., Mor G., Globerson A., Thole H., and Kohen F. (1993). Expression of estrogen receptors in thymocytes. Endocr. J. **1**: 211-217.

Besedovsky H.O., and del Rey A. (1996). Immune-neuro-endocrine interactions: Facts and hypotheses. Endocr. Rev. **17**: 64-102.

Clarke A.G., and Kendall M.D. (1994). The thymus in pregnancy: The interplay of neural, endocrine and immune influences. Immunol. Today **15**: 545-551.

Cutolo M., Sulli A., Seriolo B., Accardo S., and Masi A.T. (1995). Estrogens, the immune response and autoimmunity. Clin. Exp. Rheumatol. (Italy) **13**: 217-226.

Danel L., Souweine G., Monier J., and Saez S. (1983). Specific estrogen binding sites in human lymphoid cells and thymic cells. J. Ster. Biochem. **18**: 559-563.

Eren R., Zharhary D., Abel L., and Globerson A. (1988). Age-related changes in the capacity of bone marrow cells to differentiate in thymic organ cultures. Cell. Immunol. **112**: 449-455.

Fridkis-Hareli M., Sharp A., Abel L., and Globerson A. (1991). Thymocyte development in an *in vitro* constructed chimera of irradiated fetal thymus and lymphohemopoietic cells. Thymus **18**: 225-235.

Globerson A. (1994). Thymocyte progenitors in ageing. Immunol. Lett. **40**: 219-224.

Globerson A. (1995). T lymphocytes and aging. Int. Arch. All. Immunol. **107**: 491-497.

Greene G., Gilna P., Waterfield M., Baker A., Hort Y., and Shine J. (1986). Sequence and expression of human estrogen receptor complementary DNA. Science **231**: 1150-1154.

Grossman C.J. (1985). Interactions between the gonadal steroids and the immune system. Science **227**: 257-261.

Grossman C.J. (1989). Possible underlying mechanisms of sexual dimorphism in the immune response, fact and hypothesis. J. Ster. Biochem. **34**: 241-251.

Gülino A., Screpanti I., and Pasqualini J.R. (1983). Estrogen and antiestrogen effects on different lymphoid cell populations in the developing fetal thymus of guinea pig. Endocrinology **113**: 1754-1762.

Gulino A., Screpanti I., Torrisi M., and Frati L. (1985). Estrogen receptors and estrogen sensitivity of fetal thymocytes are restricted to blast lymphoid cells. Endocrinology **117**: 47-54.

Jenkinson E.J., Franchi L.L., Kingston R., and Owen J.J.T. (1982). Effect of deoxyguanosine on lymphopoiesis in the developing thymus rudiment *in vitro*: Application in the production of chimeric thymus rudiments. Eur. J. Immunol. **12**: 583-587.

Luster M.I., Hayes H., Korach K., Tucker A., Dean J., Greenlee W., and Boorman G. (1984). Estrogen immunosuppression is regulated through estrogenic responses in the thymus. J. Immunol. **133**: 110-116.

Marchetti B., Morale C.M., Gallo F., Batticane N., Farinella Z., and Cioni M. (1995). Neuroendocrineimmunology (NEI) at the turn of the century: Towards a molecular understanding of basic mechanisms and implications for reproductive physiopathology. Endocrine **3**: 845-861.

Marchetti P., Scambia G., Reiss N., Kaye A.M., Cocchia D., and Iacobelli S. (1984). Estrogen-responsive creating-kinase in the reticulo-epithelial cells of rat thymus. J. Ster. Biochem. **20**: 835-839.

Mor G., Amir-Zaltsman Y., Barnard G., and Kohen F. (1992). Characterization of an antiidiotypic antibody mimicking the actions of estradiol and its interaction with estrogen receptors. Endocrinology **130**: 3633-3640.

Parker M.G. (1993). Structure and function of the oestrogen receptor. J. Neuroendocrinol. **5**: 223-228.

Reisner Y., Linker-Israeli M., and Sharon N. (1976). Separation of mouse thymocytes into two subpopulations by the use of peanut agglutinin. Cell. Immunol. **25**: 129-134.

Smith E.P., Boyd J., Frank G.R., Takahashi H., Cohen R.M., Specker B., Williams T.C., Lubahn D.B., and Korach K.S. (1994). Estrogen resistance caused by a mutation in the estrogen-receptor gene in a man. N. Engl. J. Med. **331**: 1056-1061.

Sömjen D., Amir-Zaltsman Y., Gayer B., Mor G., Jaccard N., Weisman Y., Barnard G., and Kohen F. (1995). Anti-idiotypic antibody as an estrogen mimetic: Removal of $F_c$ fragment converts agonist to antagonist. J. Endocrinol. **145**: 409-416.

Sthoeger Z.M., Chiorazzi N., and Lahita R.G. (1988). Regulation of the immune response by sex hormones. I. *In vitro* effects of estradiol and testosterone on pokeweed mitogen-induced human B cell differentiation. J. Immunol. **141**: 91-98.

Thole H.H., and Jakob F. (1993). Characterization of five monoclonal antibodies raised against domain E of the porcine receptor. Exp. Clin. Endocrinol. **101**: 112-118.

Thole H.H., Jungblut P., and Jakob F. (1991). The proton-driven dissociation of oestradiol-receptor dimers as a preparative tool. Biochem. J. **276**: 709-714.

Weusten J.J.A.M., Blankenstein M.A., Gmeling-Meyling F.H.J., Schuurman H.J., Kater L., and Thijssen J.H.H. (1986). Presence of oestrogen receptors in human blood mononuclear cells and thymocytes. Acta Endocrinol. **112**: 409-414.

